# The League of Mercy

**Published:** 1904-12-31

**Authors:** 


### HOSPITAL ADMINISTRATION.

### CONSTRUCTION AND ECONOMICS.

### THE LEAGUE OF MERCY.

The annual meeting of the presidents this year, which we briefly reported last week, will be memorable for its enthusiasm and interest. There is an atmosphere about the League of Mercy meetings which quickens the circulation, and makes all present feel that they are taking part in a work which is alive, for it appeals to them one and all. Another feature of these meetings is the excellent speeches which have been made by ladies. Mrs. Harrison's address last year, and Mrs. Moreing's this year, as well as Lady Somerset's speech, really put the men on their mettle, and proved the good which underlies the League of Mercy movement by its influence upon those who attach themselves to it.

The League unites everybody from the highest to the lowest. Two royal Princesses joined the movement at a meeting held at the Speaker's House, Westminster, in July 1903. The work they have accomplished is remarkable. H.R.H. Princess Victoria of Schleswig-Holstein became President of the Berkshire branch of the League, and in one year she has raised the contributions from £16 to £777, so that it is possible that next year Berkshire may proudly take its place with South Kensington at the head of the list. £1,200 is the maximum originally fixed as the contribution of any single division, and this maximum South Kensington has attained first, Fulham being close behind; whilst Berkshire promises to be the first of the Home counties to win the Blue Ribbon of the League of Mercy. H.R.H. Princess Alice of Albany, now Princess Alexander of Teck, undertook to be lady president of the Epsom—Esher and of the Kingston—Surbiton divisions of Surrey on joining the League. It was her first responsibility on being grown up. How her Royal Highness grasped this responsibility and made those under her realise theirs events have shown, for the contributions from the Epsom—Esher division have increased from £34 in 1902 to £477 in 1904, and from the Kingston—Surbiton division to £497. These results may be taken as examples of the general progress made during 1904, and we congratulate everybody concerned upon the success achieved and the enthusiasm which has been aroused in so many directions.

But the League of Mercy attracts all classes. Here is an instance of its effect on some of the humblest workers. An old woman, a rag and bone dealer, who moves about the streets a good deal in the course of her daily occupation, became a member of the League, and has worked hard and successfully during the last three years. On paying over the sums she had received to her vice-president this year, she opened her dress and showed that she had stitched her card of membership inside it over her heart. She explained that she had done this because, as she got older, she feared being run over in the streets, and she hoped, if she was ever taken to a hospital, that the kind gentlemen there would see her card of membership, and understand that when she was well she had done her best to help the hospitals and the sick.

That the League of Mercy is winning its way steadily is proved by the contributions it has made to King Edward's Hospital Fund, viz.: in 1899, £1,000; in 1900, £6,000; in 1901, £7,000; in 1902, £8,000; in 1903, £10,000; and in 1904, £14,000. Apart altogether from the direct benefit thus conferred upon the hospitals, the League of Mercy is doing excellent service by bringing an interest into the lives of a great number of people, which grows more absorbing each year. There is inspiration in the realisation of the privilege of rendering personal service in the days of health in the cause of the sick. One of the presidents, writing of this influence, dwells upon "the kind and willing co-operation of the vice-presidents and members, which have produced so great a success and made me glad and happy." Wherever the greatest success has been achieved, that success has been mainly due to the love the League of Mercy has inspired in the workers. This is the right spirit, and as it extends and grows, we have little doubt that the shilling fund, which the League was organised to create, will ultimately become the strongest source of revenue to the King's Hospital Fund.

